# An ecological connectivity dataset for Black Sea obtained from sea currents

**DOI:** 10.1016/j.dib.2024.111268

**Published:** 2024-12-31

**Authors:** Nikolaos Nagkoulis, Christos Adam, Ioannis Mamoutos, Stelios Katsanevakis, Antonios D. Mazaris

**Affiliations:** a Department of Ecology, School of Biology, Aristotle University of Thessaloniki, Thessaloniki, Greece; b Department of Marine Sciences, University of the Aegean, 81100 Mytilene, Greece

**Keywords:** Graph theory, Species, Distribution, Marine, Conservation

## Abstract

Incorporating ecological connectivity into spatial conservation planning is increasingly recognized as a key strategy to facilitate species movements, especially under changing environmental conditions. However, obtaining connectivity data is challenging, especially in the marine realm. Sea currents are essential for exploring marine structural connectivity, but transforming sea current data into spatial connectivity matrices involves complex and resource-intensive processing steps to ensure accuracy and usability. Here, an applied a graph-based methodology has been developed to transform current data into formats suitable for delineating ecological corridors and applied to Black Sea. The dataset produced can be integrated to spatial conservation prioritization tools to incorporate connectivity in the analysis. This approach involved converting current centroids into points and projecting current directions and magnitudes onto a nearest-neighbour graph connecting these points. Using open-source data from the Copernicus Black Sea Physics Reanalysis dataset from 1993 to 2023, a high-resolution dataset of graph objects (edge lists) and shapefiles (points and edges) for the Black Sea has been created. Analyses were conducted in R, and the algorithm developed to produce the data is accessible on Zenodo. The resulting datasets are compatible with multiple software platforms (e.g., R, Python, and QGIS). A total of 17 datasets are provided from 1993 to 2023: twelve for monthly, four for seasonal, and one for yearly aggregation, supporting diverse spatial and temporal analysis needs. Overall, the datasets can be used to analyse connectivity patterns across the entire Black Sea or focus on specific regions, particularly useful for ecological modelling, and environmental protection purposes.

Specifications Table

**Table:** 

Subject	Environmental Science / Ecology
Specific subject area	A marine connectivity dataset for ecological conservation
Type of data	graph objects: edge lists (.csv X 17): processed spatial points and lines (shapefiles X 17): processed
Data collection	The input data were obtained from Copernicus Black Sea Physics Reanalysis data from 1993 to 2023. The data were then aggregated monthly, seasonally and yearly and normalized. A grid of points was created, and nearest neighbour graphs were created by connecting nearing points. Finally, the current values were projected to the graph resulting in a directed weighted nearest neighbour graph. The outputs of the analysis were exported in various formats to fulfil potential user needs.
Data source location	The location of the data is Black Sea, longitude: from 27.351 to 41.968, latitude: from 40.845 to 46.818, EPSG:4326 - WGS 84
Data accessibility	Repository name: Zenodo Data identification number: 10.5281/zenodo.14171004 Direct URL to data: 10.5281/zenodo.14171004 The code developed to create the dataset is available at 10.5281/zenodo.14501021
Related research article	None

## 1 Value of the Data

• The integration of ecological connectivity into conservation planning has evolved from a scientific concept to a key policy objective (e.g., United Nations’ 2030 Agenda for Sustainable Development Targets 2 and 3, European Biodiversity Strategy for 2030). This dataset contributes to this policy priority in the particularly challenging marine context by facilitating the identification of ecological corridors and barriers, assessments of connectivity patterns, and identification of habitats or sites that act as critical hubs for species and gene movement.
• The data can be used to apply classical graph-based analysis (e.g., shortest paths) and quantify graph-based metrics (e.g., centralities) to identify locations which facilitate or impede movement of marine species. These datasets can also serve as inputs for mathematical models for recognizing ecological corridors or into spatial prioritization tools (e.g., Marxan Connect, PrioritizeR) to strengthen species connectivity protection. Graph based metrics, such as betweenness and eigenvector centrality, that can be directly generated can enable the identification of critical habitats that support broader connectivity, facilitating the strategic allocation of conservation efforts to mitigate fragmentation and enhance biodiversity resilience.
• Beyond biological applications, the dataset can be utilized to analyse patterns of marine litter transfer and oil pollution, providing insights into their movement and distribution.
• The dataset was generated using an adaptable algorithm (available on Zenodo) allowing users to incorporate alternative current datasets (often available at varying temporal and spatial scales) or customize transformations of the original data for specific applications such as spatial conservation and restoration prioritization, pollution monitoring, or disaster rapid response.

## 2 Background

Ecological connectivity is fundamental to conservation planning, as it accounts for the physical and biological properties of seascapes and landscapes that influence the movement of organisms and genes. Integrating ecological connectivity into spatial conservation planning frequently depends on the creation of spatial layers that reflect patterns of animal movement [1]. However, actual animal movement (i.e., functional connectivity) is often difficult to track, particularly for planktonic stages, rendering seawater movement (i.e., structural connectivity) a useful surrogate for species dispersal. Spatial prioritization tools, such as Marxan Connect [2], support connectivity prioritization by integrating graph theoretical metrics to guide conservation strategies that mitigate fragmentation and enhance biodiversity resilience [3,4].

While edge-list graph datasets (reflecting transfer from one point to another) are commonly used for connectivity assessments, they are particularly scarce for marine environments. Sea currents allow estimations of marine connectivity patterns [5], but effectively converting sea current data into edge-list formats for spatial prioritization applications remains challenging. To address this challenge, sea current data were used to create graph datasets for the Black Sea, including edge lists and shapefiles. The algorithm created to project the currents’ data to the graphs is publicly available on Zenodo, enabling users to apply custom transformations.

## 3 Data Description

The dataset consists of spatially projected graph objects designed for marine spatial conservation applications. The dataset was created using aggregated sea current data from 1993 to 2023, for the entire Black Sea. Each graph object G=(P,E,w)consists of several vectors, combined so that they create a grid of points P and directed edges E. The edge weights (w) represent the sea current magnitude at that specific location.

**Fig. 1:**
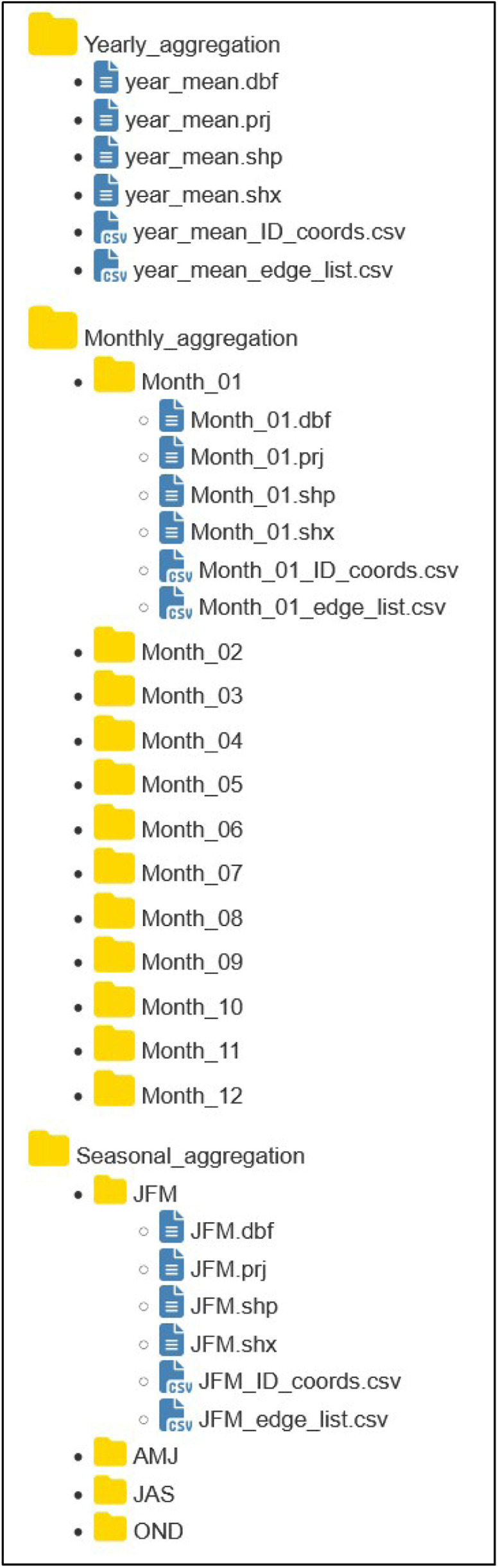
Data Repository: Three categories: Yearly aggregation (1 dataset), Monthly aggregation (12 datasets), Seasonal aggregation (4 datasets). Each dataset includes a shapefile (.dbf, .prj, .shp, .shx files), an edge list (.csv) and a coordination object (.csv)

In terms of temporal discretization, three periods are used: monthly data (12 datasets), seasonal data (4 datasets), and yearly data (1 dataset) (Fig. 1). Users can select temporal resolutions, specify time frames, and choose data formats (edge lists or shapefiles) that best fit their needs. The information provided by either format type is equivalent, however shapefiles might fit better to users interested in visualization, while edge lists are better suited for spatial analysis. In the following sections the data focusing on the edge list and the shapefile formats are presented respectively.

### 3.1 Graph objects

Edge lists represent directed connections between points. Table 1 provides an example from the yearly aggregated dataset (approx. 1,80,000 rows × 3 columns). The dataset provided in .csv format contains three columns. The first column refers to the ID of the starting Point; the second column the ID of the ending Point, and the last column represents the directed connectivity between the two points, ranging from 0 to 1, with higher values suggesting higher connectivity.

**Table 1:** The 10 first elements of the edge list (yearly aggregation).

ID start	ID end	w
1	2	0.029
3	4	0.045
5	6	0.013
7	5	0.003
8	9	0.020
10	8	0.023
11	10	0.014
12	13	0.011
14	15	0.040
16	14	0.048

Coordinates corresponding to each point ID are stored in a separate file under the GWS84 projection system (Table 2).

**Table 2:** The coordinates of the first 5 Points.

ID	x	y
1	31.11074051	46.60999528
2	31.07370349	46.60999528
3	31.18481456	46.60999528
4	31.14777754	46.60999528
5	31.25888862	46.60999528

For example, the edge $e_{8,9}$ connects the points $P_{8},P_{9}$, with a connection magnitude of $w_{8,9}=0.020$. Similarly, the edge $e_{10,8}$ connects the points $P_{10},P_{8}$, with a connection magnitude of $w_{10,8}=0.023$. While limited insights can be gained by examining these edges individually, spatially projecting the points enables detection, representation, and visualization of spatial patterns.

### 3.2 Spatial lines

**Fig. 2:**
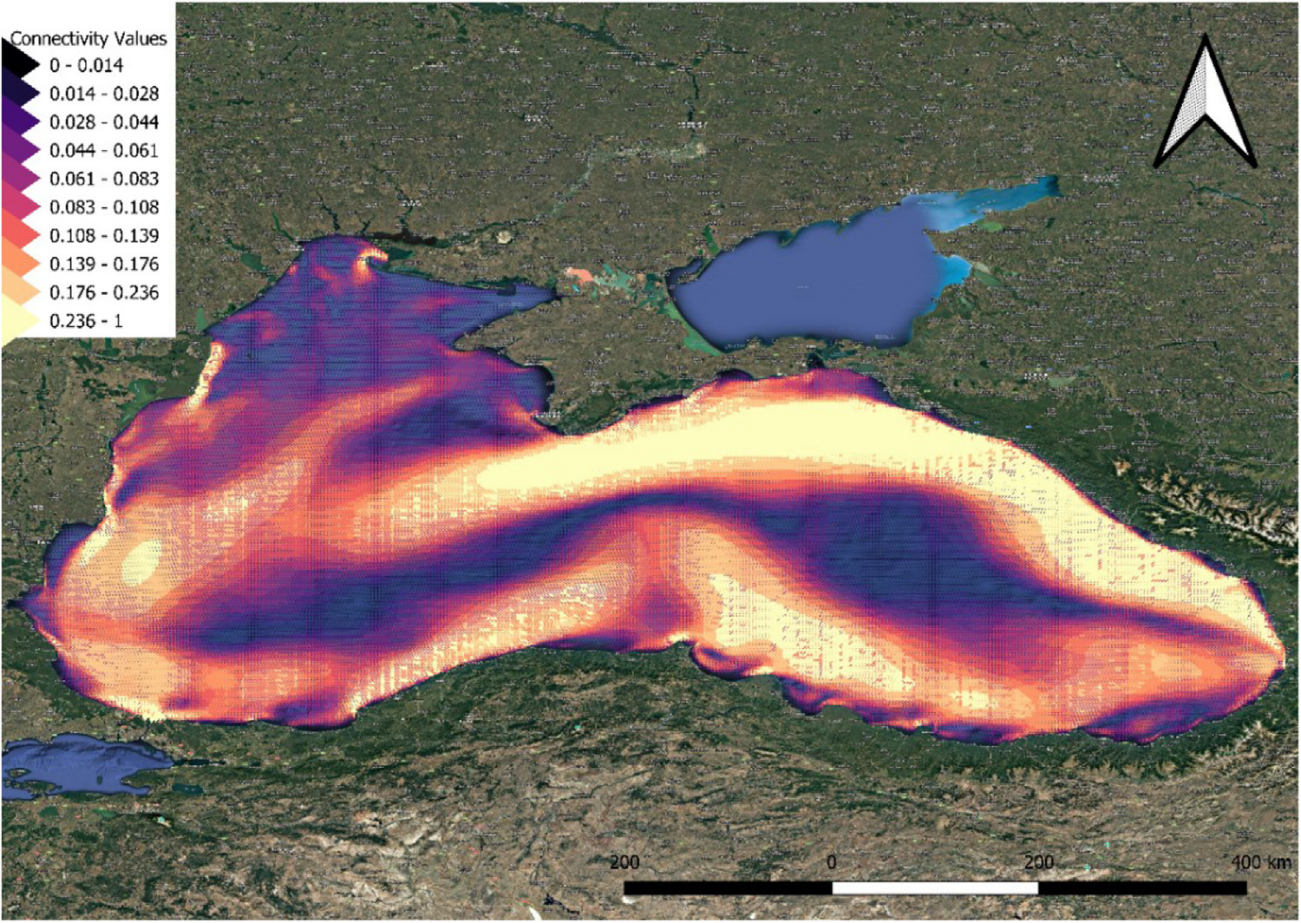
Annual aggregation shapefile for Black Sea. The data are accessed through QGIS. Higher edge weights are indicated by yellow colors and wider arrows.

**Fig. 3:**
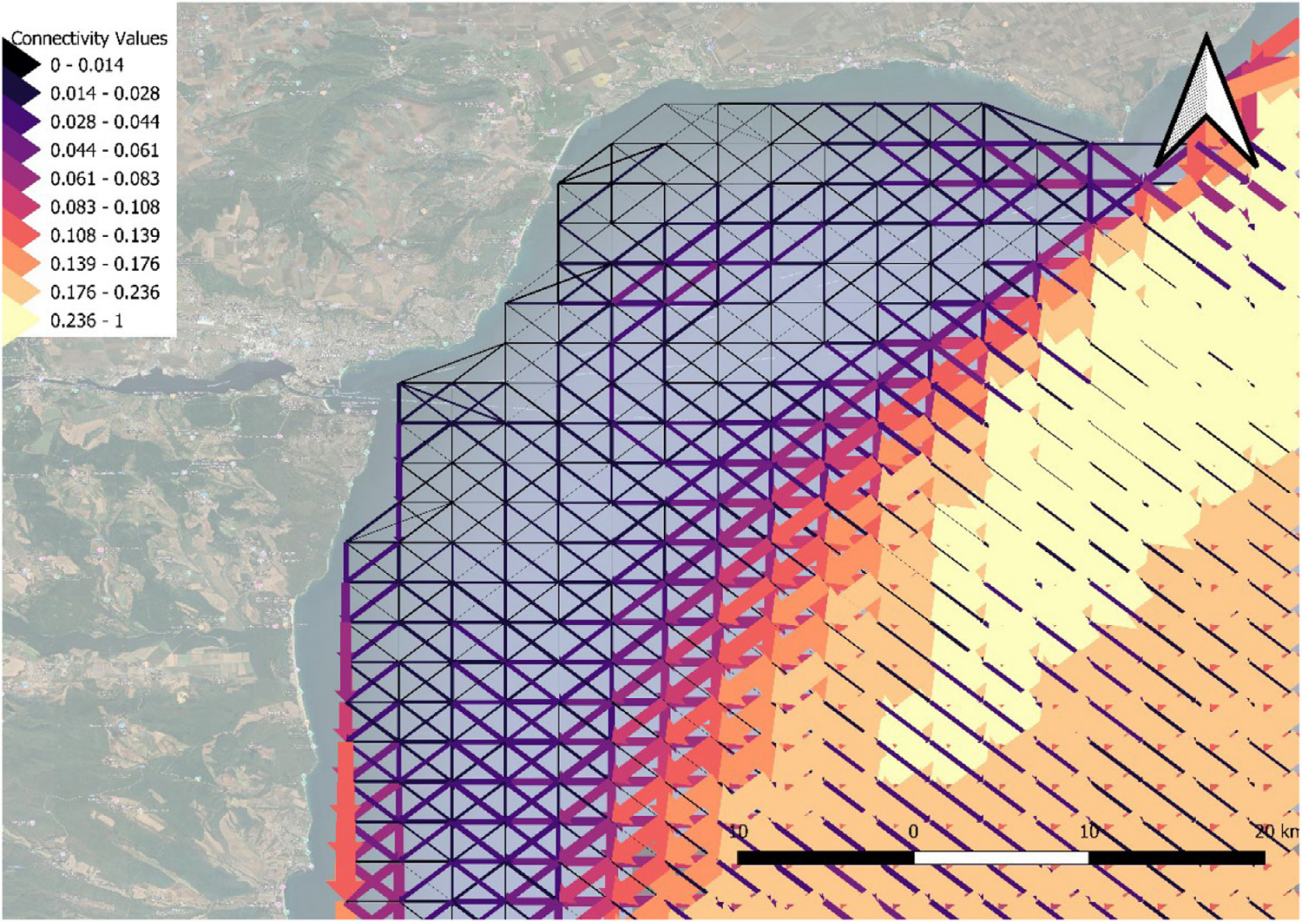
Annual aggregation shapefile for Black Sea near Varna (Bulgaria). The data are accessed through QGIS. Higher edge weights are indicated by yellow colors and wider arrows.

Another way to present the graph objects is through shapefiles, consisting of points and spatial lines. To make the data accessible to non-graph experts, shapefiles are provided to simplify visualization and processing using widely used spatial analysis software such as QGIS [6]. As an example, the yearly aggregated data is visualized across the entire region (Fig. 2) and focusing on a specific sub-region (Fig. 3) to provide more detailed insights. The yearly, seasonal, and monthly aggregated datasets are available in the aforementioned folders in the zenodo database.

In both figures, edges are represented as arrows, with each edge having a starting and ending point. These points are hidden to enhance visualization clarity. The edges connect neighboring points, forming a grid.

## 4 Experimental Design, Materials and Methods

### 4.1 Raw material preparation

The sea current data employed in this study were obtained from the Copernicus Marine Service (CMEMS) Black Sea Physics Reanalysis (product name: BLKSEA_MULTIYEAR_PHY_007_004, [7]). This dataset provides daily average 3D velocity fields (in *m/*s) at 31 unevenly spaced depth levels (from 2.5 m to 2140 m), covering the main area of the Black Sea, excluding the Azov Sea. The data span the period from January 1, 1993, to December 31, 2023, with a spatial resolution of 1/27^o^ and 1/36^o^ in the zonal and meridional directions, respectively. For this study, only the uppermost surface layer velocities from the model's vertical grid, corresponding to a depth of 2.5 m, were utilized.

A mean climatological year with daily temporal resolution over the 31-year period (01/01/1993 to 31/12/2023) was estimated using Climate Data Operators tools [8]. Additionally, four seasonal means of the climatological year were estimated: January, February, March (JFM), April, May, June (AMJ), July, August, September (JAS), and October, November, December (OND). A temporal mean for all days was also calculated.

For each of these cases, the spatio-temporal maximum velocity magnitude was estimated to normalize the velocity and its components, i.e., horizontal (*u*) and vertical (*v*) – into non-dimensional data. The following directions were considered as positive for the raster values: eastward for u and northward for v. The normalization process involved the following steps: (1) a (u,v) set was selected, (2) the magnitude m of each grid cell was calculated as $\left|\left|m\right|\right|=\sqrt{u^{2}+v^{2}\;}$, and (3) the maximum magnitude value was used to normalize both u and v.

**Fig. 4:**
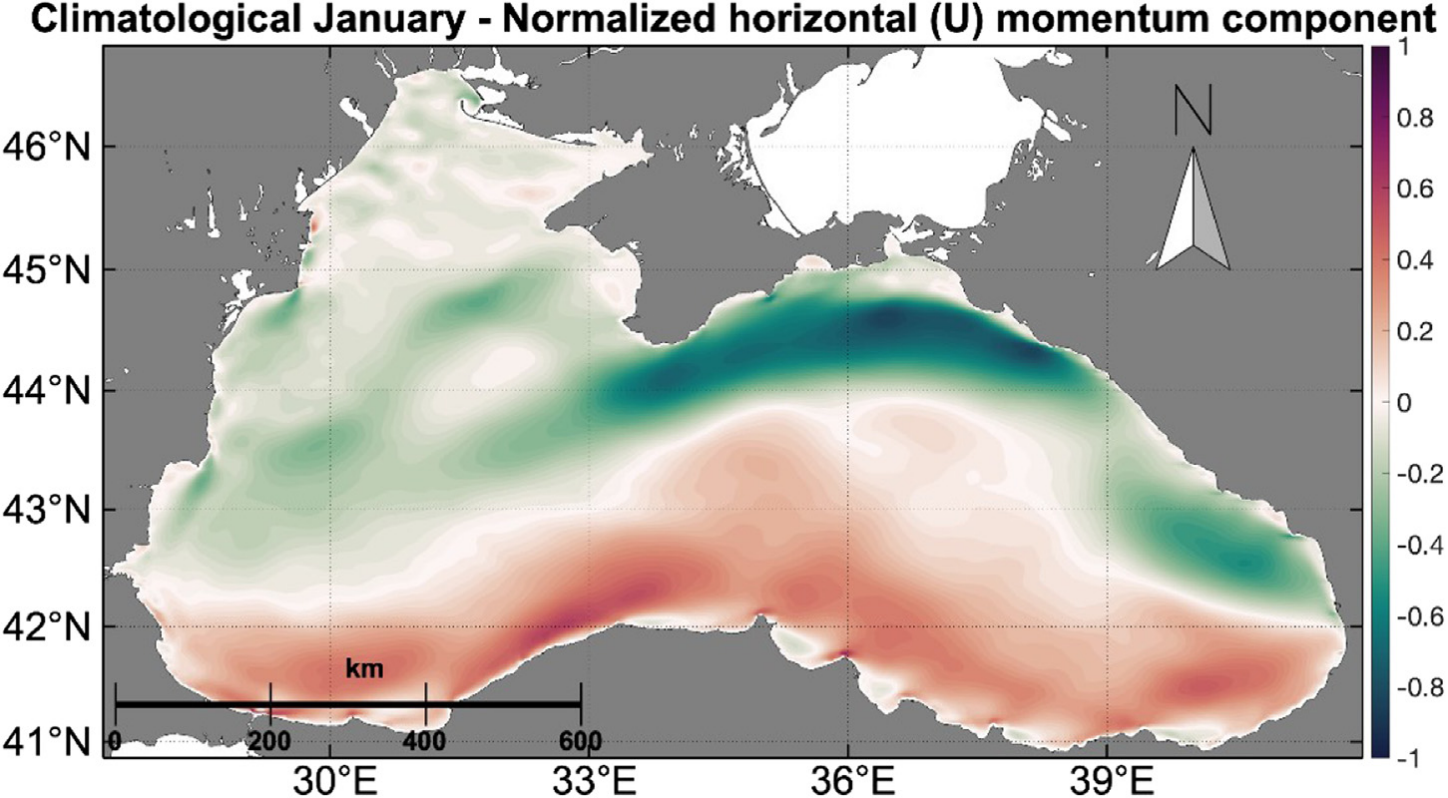
Monthly mean of normalized horizontal (*u*) momentum component for the climatological January.

An example of the normalized horizontal component (u)of the surface velocity for the climatological January is given in Fig. 4. This process resulted in 34 geotiff outputs (17 for the horizontal (*u*) direction and 17 for the vertical (*v*) direction).

### 4.2 Graphs formation

To create the connectivity datasets, it was necessary to construct weighted directed graphs. A weighted directed graph is defined as a triplet G=(P,E,w), in which P represents the set of Points (nodes / vertices), E stands for the set of directed edges, and w is a numeric value assigned to each edge. In this study, only positive values were assigned to edges, derived from the marine current u and v rasters, which served as the raw data for the analysis. The datasets were created using R, following this process for each of the 17 current datasets:

(1) Each current dataset, consisting of two rasters, one for u direction and one for v direction, was loaded into R using the terra package [9].
(2) For each pixel, centroids were extracted to create a set of points using the sf package [10].
(3) A grid of nearest neighbors was created, considering only neighboring points to create edges, thereby forming a grid of edges and points. Each point was connected to its 7 nearest neighbors using edges, which were represented as spatial lines in R.
(4) The current values were then projected onto the edges.

**Fig. 5:**
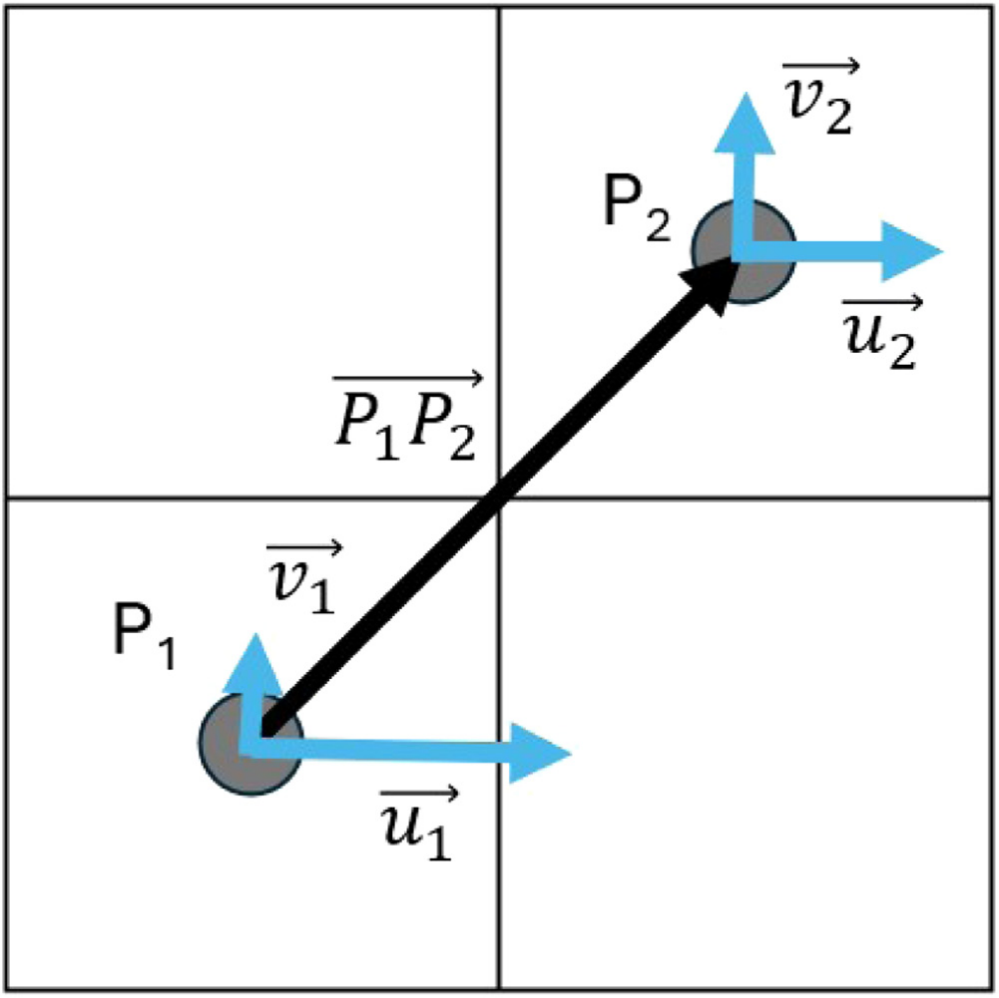
Two Neighboring Points $\left(P_{1},P_{2}\right)$ are connecting using a directed edge $\overset{⟶}{P_{1}P_{2}}$. The values $\overset{⟶}{u_{1}},\overset{⟶}{v_{1}}$ and $\overset{⟶}{u_{2}},\overset{⟶}{v_{2}}$ indicate the currents’ raster values

The projection of the u,v current values onto the edges (spatial lines) was done as follows: Suppose an edge connects a starting point $P_{1}$ $\left(x_{1},y_{1}\right)$ to an ending point $P_{2}$ $\left(x_{2},y_{2}\right)$. Each of the points $P_{1}$ and $P_{2}$ is assigned a set of current values derived from the underlying raster: $\overset{⟶}{u_{1}},\overset{⟶}{v_{1}}$ and $\overset{⟶}{u_{2}},\overset{⟶}{v_{2}}$, respectively (Fig. 5).

High absolute values indicate high current speeds, low absolute current values indicate low current speeds and the signs indicate direction. To estimate the weight of each edge, the following five step procedure was applied:

(1) A vector was created representing the edge: $\overset{⟶}{P_{1}P_{2}}=\left(x_{2}-x_{1},y_{2}-y_{1}\right)$
(2) The average current vector was computed using the average values of the two vectors: $\overset{⟶}{P_{1}{P_{2}}_{cur}}=\left(0.5\left(\overset{⟶}{u_{1}}+\overset{⟶}{u_{2}}\right),0.5\left(\overset{⟶}{v_{1}}+\overset{⟶}{v_{2}}\right)\right)$.
(3) Using the dot product, the angle between the two vectors were calculated and the $\overset{⟶}{P_{1}{P_{2}}_{cur}}$ vector was projected onto $\overset{⟶}{P_{1}P_{2}}$.
(4) The magnitude (absolute value) of the projected vector was assigned as the weight of the corresponding graph edge $\overset{⟶}{e_{w}}$.
(5) The sign of the projected vector determined the direction of $\overset{⟶}{P_{1}P_{2}}$. If the sign was positive, the direction of $\overset{⟶}{P_{1}P_{2}}$ remained unchanged, whereas if the sign was negative, the direction was reversed to $\overset{⟶}{P_{2}P_{1}}$, so that it matches the current's direction.

**Fig. 6:**
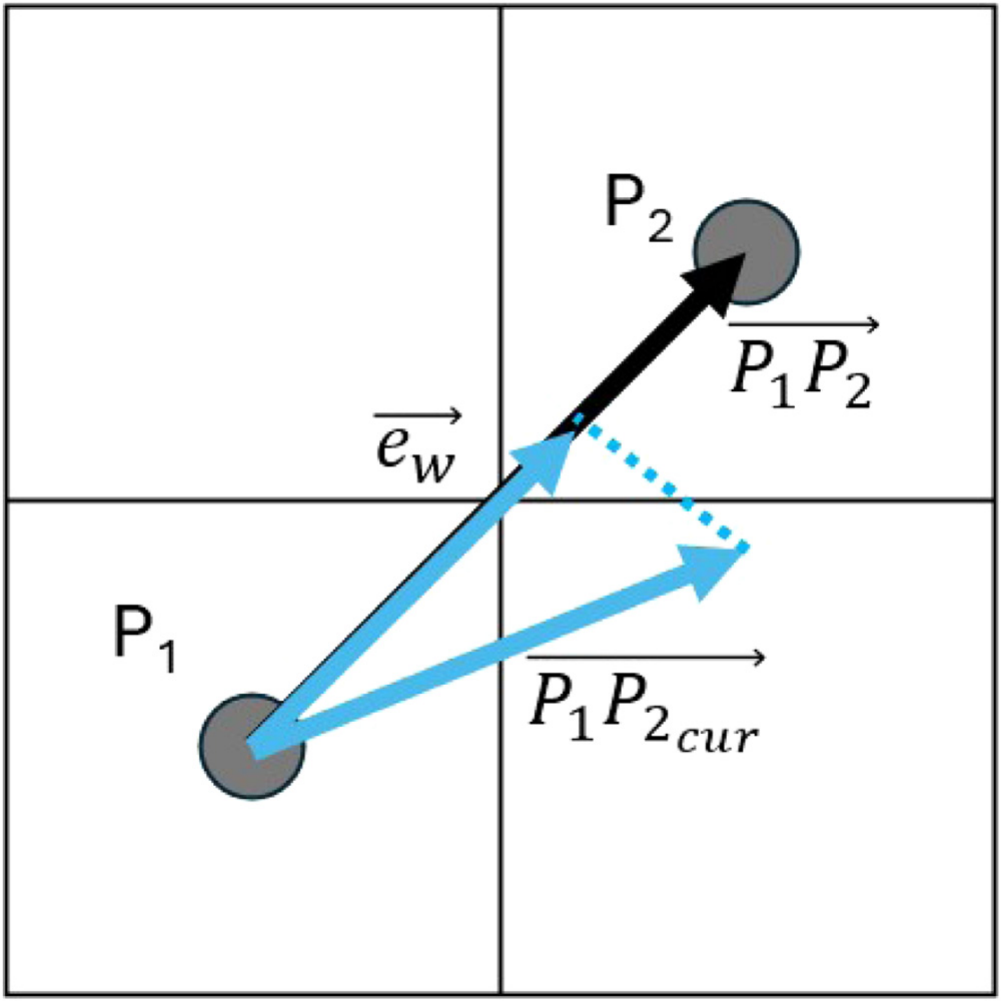
The projection of the current vector on the edge connecting $P_{1}$ and $P_{2}$. The average current vector is represented using the blue line $\overset{⟶}{P_{1}{P_{2}}_{cur}}$. The projection of the current vector onto the edge $\overset{⟶}{P_{1}P_{2}}$ is represented using the blue line $\overset{⟶}{e_{w}}$.

This method provided the graph weights for every edge (Fig. 6). The graph objects were exported as edge lists and shapefiles using the sfnetwork package [11]. This process was repeated for each one of the 17 input datasets, resulting in 17 outputs. Finally, each output was normalized using its maximum value, scaling the weights to a range of 0 to 1.

## Limitations

The findings are influenced by the limitations of the original dataset. Performing analysis in higher resolution than the resolution supported by the input Copernicus dataset can result in error. In the dataset created, the points are connected using lines segments. In very small-scale analysis, those line segments (connectivity edges) may erroneously appear over land areas. The provided connectivity datasets rely exclusively on ocean currents, which will not adequately represent ecological (functional) connectivity for species whose movement does not explicitly dependent on currents (e.g., cetaceans, sea turtles).

## Ethics Statement

Authors have read and agree to abide by the ethical requirements for publication in Data in Brief and confirm that the current work does not involve human subjects, animal testing, or data collected from social media platforms.

## Declaration of Competing Interest

The authors declare that they have no known competing financial interests or personal relationships that could have appeared to influence the work reported in this paper.

## Data Availability

ZenodoAn Ecological Connectivity dataset for Black Sea obtained from Sea Currents (Original data).
